# Adaptive Redox Response of Mesenchymal Stromal Cells to Stimulation with Lipopolysaccharide Inflammagen: Mechanisms of Remodeling of Tissue Barriers in Sepsis

**DOI:** 10.1155/2013/186795

**Published:** 2013-04-18

**Authors:** Nikolai V. Gorbunov, Bradley R. Garrison, Dennis P. McDaniel, Min Zhai, Pei-Jyun Liao, Dilber Nurmemet, Juliann G. Kiang

**Affiliations:** ^1^Radiation Combined Injury Program, Armed Forces Radiobiology Research Institute, Bethesda, MD 20889-1402, USA; ^2^Biomedical Instrumentation Center, Uniformed Services University of the Health Sciences, Bethesda, MD 20814, USA; ^3^Department of Radiation Biology, Uniformed Services University of the Health Sciences, Bethesda, MD 20814, USA; ^4^Department of Medicine, Uniformed Services University of the Health Sciences, Bethesda, MD 20814, USA

## Abstract

Acute bacterial inflammation is accompanied by excessive release of bacterial toxins and production of reactive oxygen and nitrogen species (ROS and RNS), which ultimately results in redox stress. These factors can induce damage to components of tissue barriers, including damage to ubiquitous mesenchymal stromal cells (MSCs), and thus can exacerbate the septic multiple organ dysfunctions. The mechanisms employed by MSCs in order to survive these stress conditions are still poorly understood and require clarification. In this report, we demonstrated that *in vitro* treatment of MSCs with lipopolysaccharide (LPS) induced inflammatory responses, which included, but not limited to, upregulation of iNOS and release of RNS and ROS. These events triggered in MSCs a cascade of responses driving adaptive remodeling and resistance to a “self-inflicted” oxidative stress. Thus, while MSCs displayed high levels of constitutively present adaptogens, for example, HSP70 and mitochondrial Sirt3, treatment with LPS induced a number of adaptive responses that included induction and nuclear translocation of redox response elements such as NFkB, TRX1, Ref1, Nrf2, FoxO3a, HO1, and activation of autophagy and mitochondrial remodeling. We propose that the above prosurvival pathways activated in MSCs *in vitro* could be a part of adaptive responses employed by stromal cells under septic conditions.

## 1. Introduction

It is well documented that common complications of traumatic injury and acute irradiation syndrome are bacterial infection and associated sepsis, which are considered as the major factors of high morbidity and mortality of the illnesses [1–4]. Sepsis has been defined as the acute systemic inflammatory response syndrome that occurs during infection and toxicosis [2]. Therefore, work in the field of septic shock has long focused on inflammation as the leading pathogenic mechanism. However, a variety of therapeutic approaches, mainly anti-inflammatory in nature, have failed to cure human sepsis (e.g., studies involving IL-1*α*, TNF-*β*, prostaglandins, leukotrienes, etc.) [2, 5]. Due to the failure of anti-inflammatory strategies, the physician community faces the question of whether inflammation or immunosuppression is the driving factor of death from sepsis [2, 5]. This problem leads to the search for other potential mechanisms that could produce adverse effects on host metabolome resulting in septic toxicosis. The elucidation of other major (vital) pathways affected by the oxidative stress (redox stress) from the acute bacterial inflammation is critical to solving this problem. Indeed, cumulative oxidative effects of reactive oxygen and nitrogen species (ROS and RNS, resp.) generated in overreactive responses of the reticuloendothelial, endothelial, and lymphoepithelial cells to bacteria and bacterial factors can eventually alter integrity of tissue barriers, which sustains immunochemical homeostatic interactions of tissues with internal and external environments. 

It has been well determined that one of the essential constituents of tissue barriers is mesenchymal stromal cells (MSCs) [6–9]. Although MSCs are considered to be ubiquitously integrated into conjunctive, vascular, skin, lung, intestinal, and other tissues, their major source in the body is the bone marrow, which releases MSCs upon injury and inflammation [6–9]. The data obtained recently from the research conducted on bone marrow MSCs show that these cells display antibacterial and immunomodulatory properties, can moderate septic toxicosis and improve survival in experimental sepsis [10–14]. Moreover, the effector system which mediates MSCs response to inflammatory stimuli, such as LPS, is composed of a network of toll-like receptors and pattern-recognition receptors [14], for example, the molecular machinery that can also promote inflammatory redox stress [15–18]. In conjunction with these phenomena, there are numerous data gathered from various models indicating that, paradoxically, the inflammagens directly and indirectly can also induce the cellular prosurvival adaptive mechanisms mediated by the redox-response elements and autophagy [18–27]. So far, there is only limited information on the adaptive mechanisms enabled in MSCs under inflammatory conditions [14, 28]. In part, it could be due to complicity of architecture of mesenchymal network in tissues. Therefore, in the current work we explored primary cultures of mouse bone marrow MSCs challenged with lipopolysaccharide (LPS) inflammagen.

We hypothesized that (i) a challenge of MSCs with LPS could result in the redox stress; (ii) the adaptive response of MSCs to the redox stress was accompanied by upregulation of the redox-response factors such as thioredoxin-1 (Trx1), apurinic apyrimidinic endonuclease redox effector factor 1 (Ref1), nuclear factors NF*κ*B, forkhead box O3a (FoxO3a), and NF-E2-related factor 2 (Nrf2), heme oxygenase 1 (HO1), and autophagy; (iii) activation of autophagy in the LPS-challenged MSCs enabled remodeling of the damaged cellular constituents including mitochondria. The objective of this communication is to provide experimental evidence of a potential role of MSCs in sustaining redox homeostasis of tissue barriers under the septic oxidative stress.

## 2. Materials and Methods

Mouse bone marrow mesenchymal stromal cells (MSCs) phenotype and features are well defined in recent reviews [14]. The establishment of MSC cultures used in the present research was described previously [11] when they were determined to be the bone marrow colony-forming unit fibroblasts [11, 29]. They lack hematopoietic and endothelial lineage markers (CD45, CD34, CD4, and CD117) but are positive for a wide variety of other cell surface molecules (CD44, CD105, and Sca1). The cells expressed collagen type III and matrix metalloproteinases types 3, 9, and 13 and responded to stimulation with the platelet-derived growth factor. These cells were expanded and cultivated in hypoxic conditions (5% O_2_, 10% CO_2_, 85% N_2_) in Mesencult medium (Stemcell Technologies Inc.).

MSC cultures were grown to approximately 80% confluency prior to being used in the experiments. LPS (Sigma-Aldrich Co., catalog number L4391) from *E. coli* 0111:B4 was used in concentrations of 0.05–2.5 *μ*g/mL. Challenge of MSCs with LPS was conducted in a “pulse” mode for 1–3 h, and then incubation medium was replaced with a fresh one. Pyrrolidine dithiocarbamate (PDTC, 10 *μ*M) was used to inhibit NF*κ*B-mediated response to the LPS challenge as reported recently [30].

The challenged cells were either fixed or harvested and, then lysed at different time points following LPS challenge (1–24 h). The obtained cell lysates were kept frozen at −80°C until further analyses. The LPS-induced gene and protein expressions were determined by qRT-PCR and immunoblotting techniques. Fluorescence imaging techniques were used for (i) assessment of nuclear translocation of p65 subunit of NF*κ*B, that is, (p65) NF*κ*B, thioredoxin 1 (TRX1), Ref1, and nuclear factor (erythroid-derived 2)-like 2, (Nrf2), (ii) expression and activity of iNOS, (iii) assessment of formation of ROS and RNS with dihydrorhodamine 123 assay, (iv) assessment of apoptotic transformations with annexin V assay, (v) formation of LC3-containing autophagosomes and mitochondrial fusion, and (vi) estimation of proliferative activity with Ki67 marker. LPS-induced mitochondrial remodeling and mitophagy, that is, autophagy of mitochondria, were demonstrated through transmission electron microscopy (TEM). 

For qRT-PCR analyses, total cellular RNA was isolated from MSCs using the Qiagen RNeasy Miniprep kit, quantified by measuring the absorbance at 260 nm and qualified by electrophoresis on a 1.2% agarose gel. cDNA was synthesized using SuperScript II (Invitrogen) and qRT-PCR was performed using SYBR Green iQ Supermix (Bio-Rad), each according to the manufacturers' instructions. The following primer sequences were used for qRT-PCR: iNOS Forward 5′ CAGCTGGGCTGTACAAACCTT 3′; iNOS Reverse 5′ CATTGGAAGTGAAGCGTTTCG 3′; IL-1a Forward 5′ CGGGTGACAGTATCAGCAAC 3′; IL-1a Reverse 5′ GACAAACTTCTGCCTGACGA 3′; IL-1b Forward 5′ CCCAACTGGTACATCAGCAC 3′; IL-1b Reverse 5′ TCTGCTCATTCACGAAAAGG 3′; IL-6 Forward 5′ AGTCGGAGGCTTAATTACACATGTT 3′; Il-6 Reverse 5′ AAGTGCATCATCGTTGTTCATACA 3′; IL-8 Forward 5′ GCGCCTATCGCCAATGAG 3′; IL-8 Reverse 5′ AGGGCAACACCTTCAAGCTCT 3′. The quality of qRT-PCR data was verified by melt curve analysis, efficiency determination, agarose gel electrophoresis, and sequencing. Relative gene expression was calculated by the method of Pfaffl using the formula 2^−ΔΔ*Ct*^.

For protein analyses, MSCs were lysed and total proteins were extracted in accordance with the protocol described previously [11]. Aliquots of proteins were resolved on SDS-polyacrylamide slab gels (NuPAGE 4%–12% Bis-Tris; Invitrogen, Carlsbad, CA, USA). After electrophoresis, proteins were blotted onto a PDVF membrane and the blots were incubated with antibodies (1 *μ*g/mL) raised against MAP LC3, Nrf2, (p65) NF*κ*B, HSP70, Sirt3, p62/SQSTM1, HO1, iNOS, and actin (Abcam, Santa Cruz Biotechnology Inc., EMD Millipore, and Sigma-Aldrich, Co.) followed by incubation with species-specific IgG peroxidase conjugate. 

For expression and spatial localization of proteins in MSCs, the cells (5 specimens per group) were fixed in 2% paraformaldehyde, processed for immunestaining, and analyzed with fluorescence confocal microscopy [11]. Normal donkey serum and antibody were diluted in phosphate-buffered saline (PBS) containing 0.5% BSA and 0.15% glycine. Any nonspecific binding was blocked by incubating the samples with purified normal donkey serum (Santa Cruz Biotechnology, Inc., Santa Cruz, CA, USA) diluted 1 : 20. Primary antibodies were raised against MAP LC3, iNOS, Ref1, Trx1, (p65) NF*κ*B, Nrf2, FoxO3a, p53, and Tom 20 (a mitochondrial marker). That was followed by incubation with secondary fluorochrome-conjugated antibody and/or streptavidin-Alexa Fluor 610 conjugate (Molecular Probes, Inc., Eugene, OR, USA), and with Heochst 33342 (Molecular Probes, Inc., Eugene, OR, USA) diluted 1 : 3000. Secondary antibodies used were Alexa Fluor 488 and Alexa Fluor 594 conjugated donkey IgG (Molecular Probes Inc., Eugene, OR, USA). Negative controls for nonspecific binding included normal goat serum without primary antibody or with secondary antibody alone. Five confocal fluorescence and DIC images of crypts (per specimen) were captured with a Zeiss LSM 710 microscope. The immunofluorescence image analysis was conducted as described previously [12].

Analysis of nitric oxide (NO) formation in LPS-challenged MSC was as follows. DAF-FM diacetate (4-amino-5-methylamino-2′,7′-difluorofluorescein diacetate, Life Technologies Corp) was utilized for detection of NO formation in living cells 24 h after challenge with LPS (500 ng/mL). DAF-FM is essentially nonfluorescent until it reacts with NO to form a fluorescent benzotriazole. The reagent solution (5 *μ*M in PBS) was applied to the cells and a formation of the fluorescent adduct was monitored with a confocal Zeiss LSM 710 microscope. L-N^6^-(1-iminoethyl)lysine (LNIL, Sigma-Aldrich Co.), a selective inhibitor of iNOS, was used for suppression of NO production in the cells. 

Dihydrorhodamine 123 (DhRho 123, Life Technologies Corp) was utilized for detection of formation of ROS and RNS (i.e., peroxynitrite) in the cells 24 h after challenge with LPS (500 ng/mL). Dihydrorhodamine 123 is an uncharged and nonfluorescent reactive oxygen species (ROS) indicator that can passively diffuse across membranes where it is oxidized to cationic rhodamine 123 which localizes in the mitochondria and the cytoplasm and exhibits green fluorescence. The reagent solution (10 *μ*M in PBS) was applied to the cells and a formation of the fluorescent product was monitored with a confocal Zeiss LSM 710 microscope. L-N^6^-(1-iminoethyl)lysine (LNIL, Sigma-Aldrich Co), a selective inhibitor of iNOS, was used for suppression of NO release and the consequent RNS-dependent oxidation of DhRho 123 in the cells.

For transmission electron microscopy (TEM), MSCs in culture were fixed in 2% formaldehyde and 2% glutaraldehyde in PBS overnight, postfixed in 2% osmium tetroxide in PBS, dehydrated in a graduated series of ethanol solutions, and embedded in Spurr's epoxy resin. Blocks were processed as described previously [11]. The sections of embedded specimens were analyzed with a Philips CM100 electron microscope.

Statistical significance was determined using Student's *t*-test for independent samples. Significance was reported at a level of *P* < 0.05.

## 3. Results

In the first set of experiments, we assessed alterations in the MSC stress-response proteins following LPS challenge. LPS, a major component of the outer membrane of gram-negative bacteria, is considered to be a strong inflammagen. In stromal cells, LPS-induced activation of Toll-like receptor type 4 triggers a danger signal leading to nuclear translocation of NF*κ*B and subsequent upregulation of several known inflammatory mediators including iNOS producing NO [14, 26, 30]. Ultimately, LPS-induced effects can result in redox stress. 

**Figure 1:**
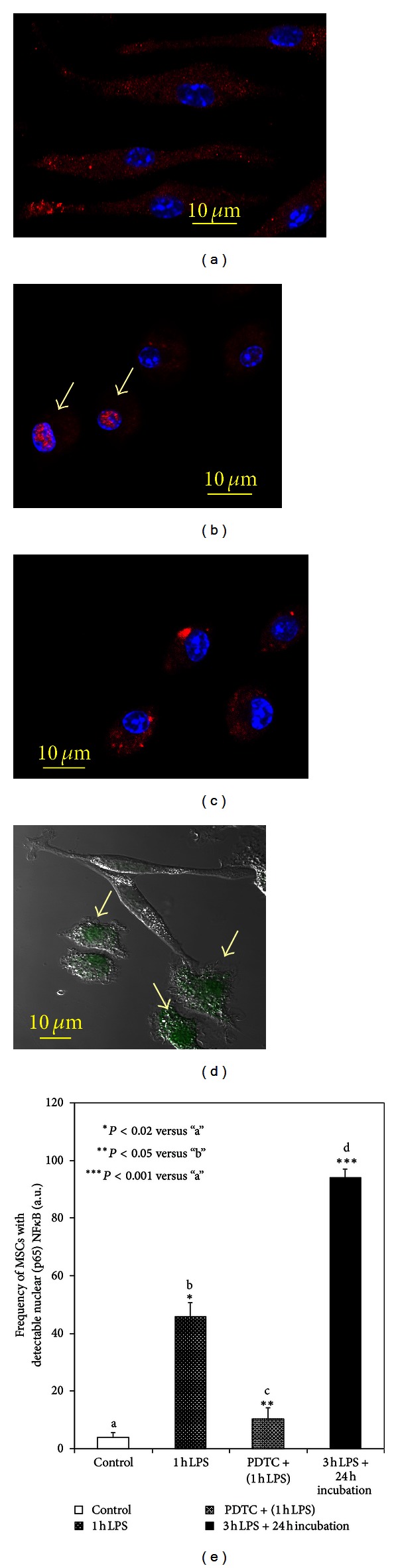
Confocal immunofluorescence assessment of nuclear translocation of (p65) NF*κ*B in MSCs following stimulation with LPS. ((a)–(c)) Overlay of projections of nuclei (blue channel) and (p65) NF*κ*B (red channel) in MSCs. (a) Control cells. (b) MSCs challenged with 500 ng/mL LPS for 1 h. Activation of (p65) NF*κ*B nuclear translocation was defined by an increase in immunofluorescence of (p65) NF*κ*B in the nuclear regions. Nuclear regions of MSCs were determined by counterstaining of nuclear DNA with Hoechst 33342 (blue channel). Nuclear localization of (p65) NF*κ*B appears in purple (indicated with arrows). (c) is the same as (b) except that MSCs were preincubated with 10 *μ*M PDTC (pyrrolidine dithiocarbamate), an inhibitor of NF*κ*B. (d) The treatments were the same as in (c) but the presented image is overlay of projections of Annexin V (green channel), a marker of proapoptotic transformations, and a respective DIC image of MSCs. Apoptotic events are indicated with arrows. The confocal images were taken with pinhole setup to obtain 0.5 *μ*m Z-sections. Conditions: MSCs were incubated with 500 ng/mL LPS in the medium either 1 h or 3 h (see Section 2). (e) Histogram depicting increase in frequency (per 100 cells) of occurrence (p65) NF*κ*B nuclear translocation in MSCs treated with LPS. Spatial appearance of (p65) NF*κ*B in MSCs was determined with immunofluorescence confocal microscopy as indicated in Section 2. Confocal projections of nuclear fractions of (p65) NF*κ*B are shown in ((a)–(c)).

As previously published, control MSCs have relatively high amounts of constitutive NF*κ*B [11]. Confocal immunofluorescence imaging of (p65) NF*κ*B revealed in controlled MSCs that the protein immunoreactivity was predominantly present in the cytoplasm (Figure 1(a)). The (p65) NF*κ*B projections in LPS-challenged cells are shown in Figure 1(b). These data suggest that the challenge of the cells with LPS promoted a prompt (within 1 h) increase in the nuclear fraction of NF*κ*B (Figure 1(b), indicated with arrows). The nuclear translocation of (p50)(p65) NF*κ*B is considered to be a part of antiapoptotic response to stress-induced factors [14, 19]. Therefore, preincubation of MSCs with 10 *μ*M PDTC, an inhibitor of the NF*κ*B pathway, suppressed nuclear translocation of (p65) NF*κ*B (Figure 1(c)) that was accompanied by proapoptotic transformations and a loss of cell confluency after application of LPS (Figure 1(d)). A summary of quantitative assessment of these LPS-induced effects is presented in a histogram in Figure 1(e). As shown in Figures 1(e) and 1(d), an increase in nuclear translocation of (p65) NF*κ*B was observed in almost all MSCs treated with 500 ng/mL LPS (3 h pulse) 24 h after-treatment. The observed LPS-induced transactivation of NF*κ*B in MSCs was accompanied by a drastic expression of proinflammatory mediators including IL-1*α*, IL-1*β*, IL-6, and iNOS that occurred in a dose-dependent manner (Table 1, qRT-PCR analysis). 

 A maximum expression of iNOS was observed at a dose of 500 ng/mL LPS (Table 1); therefore, our further experiments on LPS-induced MSC toxicity were conducted using this dose. It should be noted that while MSCs displayed resistance to substantially higher doses of LPS (up to 5000 ng/mL), they experienced inhibition of proliferative activity under those conditions (data not shown). Moreover, there is evidence (Dr. Elliott TB, unpublished data) that the LPS dose 500 ng/mL in blood can induce in mice a severe septic syndrome with a predicted mortality rate 80%–90%. 

**Figure 2:**
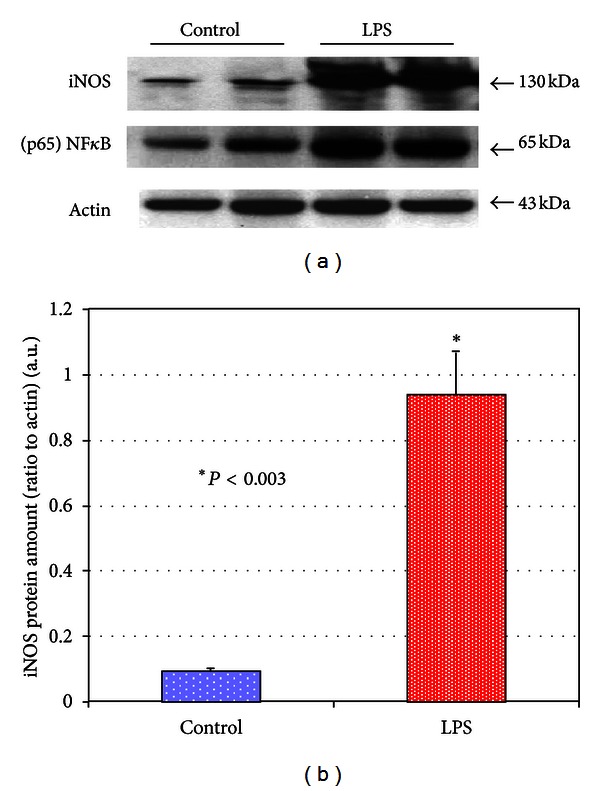
Immunoblot analysis of expression of NF*κ*B and iNOS proteins in MSCs subjected to LPS challenge. (a) Immunoblotting bands of NF*κ*B and iNOS. (b) Densitometry histograms of iNOS bands in MSCs subjected to challenge with LPS. The presented bars indicate the relative density of iNOS protein (normalized to density of actin bands). The statistical significance was determined by Student's *t*-test (*n* = 3). Conditions: MSCs were incubated with 500 ng/mL LPS for 3 h. The cells were harvested at 24 h following challenge with LPS.

**Figure 3:**
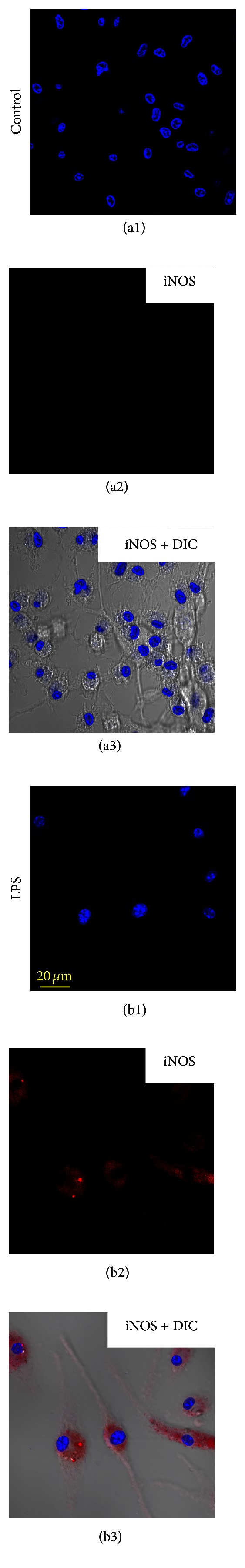
Confocal immunofluorescence imaging of iNOS protein in MSCs challenged with LPS. (a1) Projections of nuclei (blue channel) in control MSCs. (a2) Projections of iNOS (red channel), (a3) overlay of projections presented in (a1) and (a2) and a respective DIC image. (b1) Projections of nuclei (blue channel) in LPS-challenged MSCs. (b2) Projections of iNOS (red channel) and (b3) overlay of projections presented in (b1) and (b2) and a respective DIC image. A massive accumulation of iNOS occurred in the LPS-challenged MSCs. Counterstaining of nuclei was with Hoechst 33342 (blue channel). The confocal images were taken with pinhole setup to obtain 0.5 *μ*m Z-sections. Conditions were the same as indicated in Figure 2.

**Figure 4:**
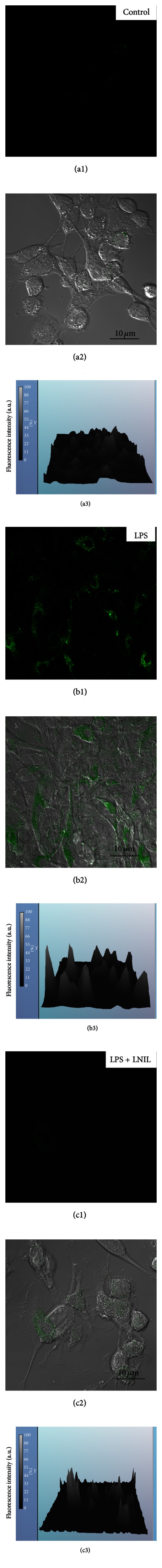
Confocal immunofluorescence imaging of the DAF-FM—detectable nitric oxide in MSCs challenged with LPS. (a1) Projection of adduct of DAF-FM with NO (DAF-FM-NO) (green channel) in control MSCs. (a2) Overlay of projection of DAF-FM-NO shown in (a1) and a respective DIC image. (a3) Histogram of relative fluorescence of DAF-FM-NO shown in (a1). (b1) Projection of adduct of DAF-FM-NO (green channel) in LPS-challenged MSCs. (b2) Overlay of projection of DAF-FM-NO shown in (b1) and a respective DIC image. A dramatic increase in DAF-FM-NO fluorescence occurred in the LPS-challenged MSCs. ((c1)–(c3)) Same as (b1–b3) except that LPS-challenged MSCs were treated with LNIL, an iNOS inhibitor. Suppression of DAF-FM-NO fluorescence occurred in the LPS-challenged MSCs.

**Figure 5:**
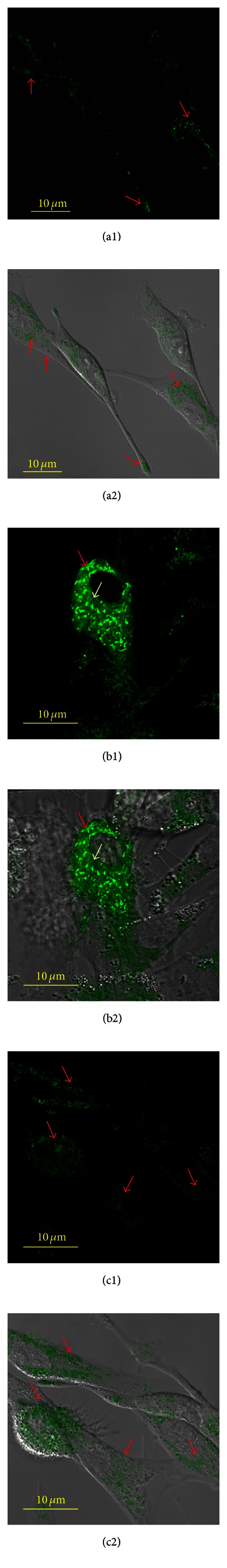
Confocal immunofluorescence imaging of the DhRho 123 detectable ROS/RNS products in MSCs challenged with LPS. (a1) Projection of oxidized form of DhRho 123 (Rho 123) (green channel) in control MSCs. (a2) Overlay of projection of Rho 123 shown in (a1) and a respective DIC image. (b1) Projection of Rho 123 (green channel) in LPS-challenged MSCs. (b2) Overlay of projection of Rho 123 shown in (b1) and a respective DIC image. A dramatic increase in Rho 123 fluorescence occurred in the LPS-challenged MSCs. (c1) Projection of Rho 123 (green channel) in LPS-challenged MSCs and treated with LNIL, an iNOS inhibitor. (c2) Overlay of projection of Rho 123 shown in (c1) and a respective DIC image. Suppression of Rho 123 fluorescence occurred in the LPS-challenged MSCs. Bright green fluorescence of the ROS-activated Rho 123 shown in mitochondria is shown with red arrows. Diffused green fluorescence of the ROS/RNS-activated Rho 123 in the cytoplasm is shown with white arrows in (b). The confocal images were taken with pinhole setup to obtain 0.5 *μ*m Z-sections. The experimental conditions were the same as indicated in Figure 2.

LPS-pulse challenge for 3 h resulted in prolong changes in redox-status of MSCs. Thus, 24 h after the LPS challenge we observed increases in (p65) NF*κ*B translocation in *≈*90% of the treated MSCs (Figure 1(e)) that was accompanied by a dramatic accumulation of iNOS protein in the cells (Figure 2). The immunoblotting data presented in Figure 2 were confirmed by immunofluorescence imaging of iNOS protein in the cells (Figure 3). Accumulation of iNOS resulted in excessive production of NO in the cells determined by increase in fluorescence intensity of the fluorescent adduct of DAF-FM with NO in the LPS-treated cells (Figures 4(a) and 4(b)). This effect was suppressed in the presence of LNIL, a specific iNOS inhibitor (Figure 4(c)). An increase in the formation of ROS and RNS associated with the LPS stimulation was monitored with DhRho 123, another molecular probe, which is subjected to oxidation in the presence of ROS and peroxynitrite and is thus converted to fluorescent Rho 123. The results of DhRho imaging in the cells are presented in Figure 5. As shown in the figure, in control cells a moderate Rho 123 fluorescence appeared only in mitochondria, a major generator of ROS under normal condition (Figure 5(a)). Dramatic changes in Rho 123 fluorescence were observed in MSCs after treatment with LPS (Figure 5(b)). Indeed, compare to control group these MSCs were characterized by substantially increased fluorescence of Rho 123 in mitochondria (Figures 5(c) and 5(d)). Moreover, green fluorescence of Rho 123 also occurred in the entire cytoplasm (Figures 5(c) and 5(d)). This observation indicated that in LPS-treatment of MSCs also caused induction of the ROS/RNS generating pathways outside the mitochondrial bodies and resulted in redox stress. Interestingly, elongation of mitochondria due to activation of mitochondrial fusion was observed under these conditions (Figure 5(b)). This increase in Rho 123 fluorescence was suppressed in the presence of LNIL, a specific iNOS inhibitor (Figure 5(c)). Overall, the presented data suggested that the LPS-challenged cells experienced the redox stress due to increase in iNOS-dependent production of NO. Therefore, we expected upregulation of the redox-response elements mediating cell adaptation to long-lasting stress conditions. 

It is well accepted that numerous vitagenes are evolutionarily adapted by cells to manage oxidative stress; they include but are not limited to redox-sensitive transcriptional factors, antioxidants, heat shock proteins, and regulators of autophagy and mitochondrial functions [22, 24, 25, 27, 31–37]. From our results presented in Figure 2, a 3 h pulse challenge of MSCs with LPS resulted in a substantial increase in NF*κ*B in the cell protein fraction. NF*κ*B is known as a redox-sensitive transcription factor that contains a critical cysteine residue (Cys-62) in the p50 subunit that is involved in DNA binding [24, 36]. NF*κ*B is normally present in the cytoplasm in a complex with the inhibitory subunit IkB but under oxidative conditions, IkB is phosphorylated by I-kB kinase (IKK), ubiquitinated, and subsequently degraded. Excessive oxidative stress can lead to the oxidation of Cys-62 which does not affect its translocation to the nucleus but rather interferes with DNA binding and decreases gene trans-activation [24, 38]. Therefore, in the presence of oxidative stress, nuclear translocation of activated (p50)(p65) NF*κ*B has to be synchronized with increase in nuclear fraction of reductants TRX1 and Ref1 [24, 38]. Overall, while the NF*κ*B system has been recognized to be primarily activated by inflammagens (such as LPS) via Toll-like and other receptors, it was the first mammalian transcription factor determined to be redox regulated and suggested to be directly activated by ROS and RNS [24, 36, 38]. 

**Figure 6:**
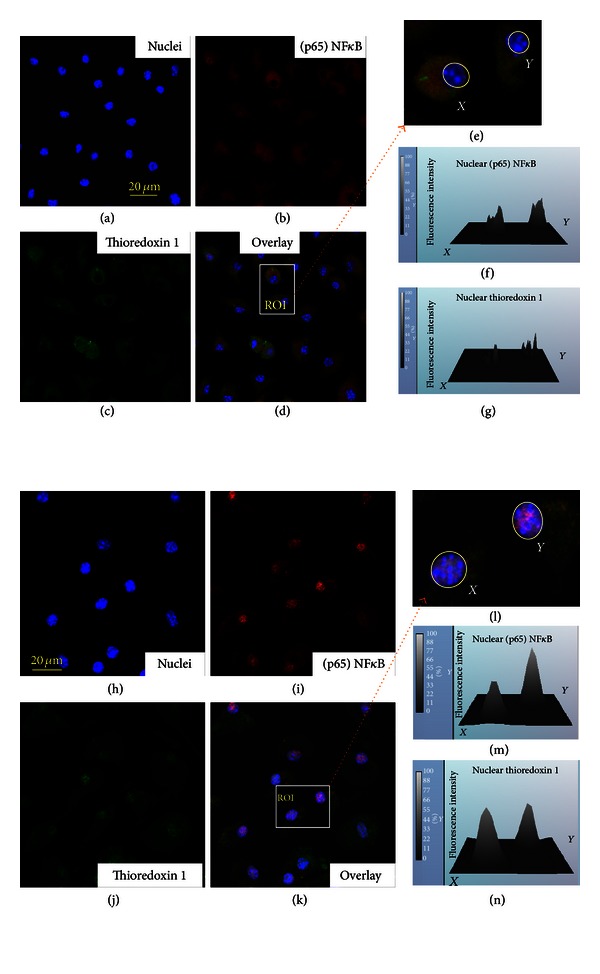
Confocal immunofluorescence imaging of nuclear translocation of NF*κ*B and thioredoxin 1 (TRX1) in MSCs challenged with LPS. ((a)–(c)) Projections of (p65) NF*κ*B (red channel) and TRX1 (green channel) in control MSCs. (d) Overlay of projections of (p65) NF*κ*B, TRX1, and nuclei presented in ((a)–(c)). (e) ROI selected in (d). (f) Histogram of immunofluorescence of (p65) NF*κ*B in nuclei shown in (e). (g) Histogram of immunofluorescence of TRX1 in nuclei shown in (e). ((h)–(j)) Projections of (p65) NF*κ*B (red channel) and TRX1 (green channel) in LPS-challenged MSCs. (k) Overlay of projections of (p65) NF*κ*B, TRX1, and nuclei presented in ((h)–(j)). (l) ROI selected in (k). ((m)-(n)) Histograms of immunofluorescence of (p65) NF*κ*B and TRX1 in nuclei shown in (l). Counterstaining of nuclei was with Hoechst 33342 (blue channel). The confocal images were taken with pinhole setup to obtain 0.5 *μ*m Z-sections. The experimental conditions were the same as indicated in Figure 2.

Confocal projections of cellular NF*κ*B shown in Figures 6(a)–6(f) indicate a relatively low level of nuclear fraction of NF*κ*B in control cells. This balance dramatically changed after challenge with LPS (Figures 6(h)–6(n)). That was associated with increases in nuclear fractions of TRX1, a reducing factor essential for activation of oxidized nuclear NF*κ*B; note that the localization of nuclear TRX1 appeared in close proximity with nuclear (p65) NF*κ*B (Figure 6). 

**Figure 7:**
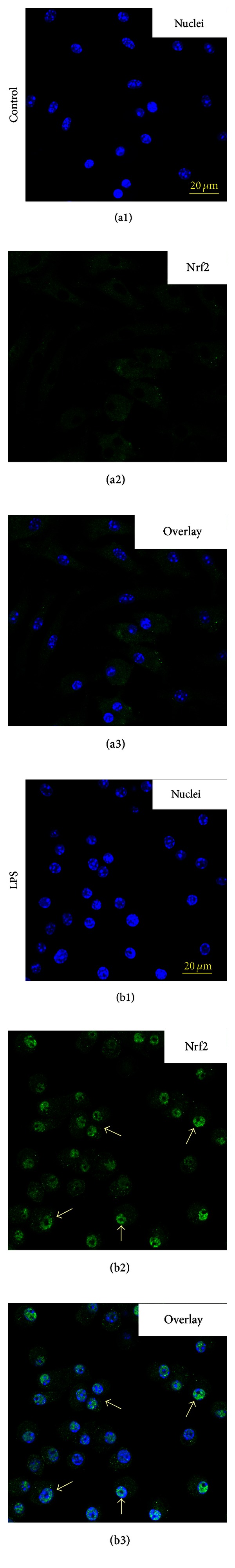
Assessment of nuclear translocation of Nrf2 redox-response element in MSCs challenged with LPS. Counterstaining of nuclear DNA was with Hoechst 33342 (blue channel). Nrf2 staining is in green. Nrf2 localized in nuclei appears in turquoise/green color due to interference of “green” and “blue” (indicated with arrows). Experimental conditions were the same as indicated in Figure 2.

One of the most crucial cellular defense mechanisms against oxidative stress and nitrosative stress is mediated by the transcription factor Nrf2 [23, 24, 33, 38, 39]. Under the basal condition, Nrf2 is compartmentalized in the cytoplasm and Nrf2-dependent transcription is repressed by a negative regulator Keap1. In the presence of ROS and RNS, Nrf2 is released from a complex with Keap1 and translocated to the nucleus where it activates antioxidant responsive element-(ARE-) dependent gene expression to maintain cellular redox homeostasis [23, 24, 40]. In this respect, mechanisms of “sensing” the redox stress by Nrf2 seem similar to those demonstrated for NF*κ*B. Therefore, it was not surprising that the patterns of upregulation of Nrf2 and NF*κ*B in the LPS-challenged MSCs were similar (Figures 2, 6, and 7), with exception that in contrast to NF*κ*B we did not observe a significant increase in nuclear fraction of Nrf2 at 1 h pulse LPS challenge of the cells (data not shown) as we did with NF*κ*B. 

**Figure 8:**
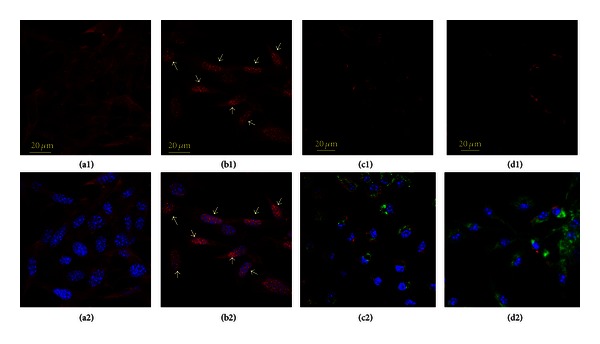
Assessment of nuclear translocation of FoxO3a and p53 redox-response element in MSCs challenged with LPS. Counterstaining of nuclear DNA was with Hoechst 33342 (blue channel). FoxO3a staining is in red (control (a1) and (a2) versus LPS treatment (b1) and (b2)). ((a2)-(b2)) overlay of confocal projections of FoxO3a protein (red channel) and nuclear DNA (blue channel). FoxO3a nuclear translocation is indicated with arrows in (b1) and (b2), where nuclear fraction of FoxO3a appears in pink color due to interference of “red” and “blue”. ((c1), (c2), (d1), (d2)) Confocal projections of p53 protein (red channel) in MSCs (control (c1) and (c2) versus LPS (d1) and (d2)). Counterstainings were done with Hoechst 33342 (for nuclear DNA, blue channel) and anti-TOM20 IgG (for mitochondria, green cannel). (c2) and (d2) are overlay of confocal projections of p53 protein (red channel), TOM20 protein (green channel), and nuclear DNA (blue channel). Note that there was no detectable increase in the p53-immunofluorescence in nuclear areas of LPS-challenged MSCs. The confocal images were taken with pinhole setup to obtain 0.5 *μ*m Z-sections. Experimental conditions were the same as indicated in Figure 2.

At this stage, it was reasonable to assume that deep metabolic changes essential for long-term survival of the cells under redox stress conditions may proceed via a cascade of events which are driven by synchronous activation (or suppression) of different signaling mechanisms. For example, we tested LPS-induced nuclear translocation of two other transcriptional factors, namely, FoxO3a and p53. FoxO3a, a member of a family of mammalian forkhead transcription factors of the class O, has been recently proposed as mediator of diverse physiologic processes, including regulation of resistance to redox stress and increase in longevity [31, 32]. Opposite to FoxO3a, p53 transcriptional factor is the well-discussed master regulator of apoptotic cell death, which can be activated in stromal cells by redox genotoxic stress [41]. The effects of LPS challenge on nuclear translocation of FoxO3a and p53 are shown in Figure 8. Indeed, as expected, a massive increase in nuclear fractions of FoxO3a occurred in the LPS-treated cells (Figure 8; control (a1) and (a2) versus LPS treatment (b1) and (b2)) that corroborated with effects observed for Nrf2 and NF*κ*B (Figures 6 and 7). Meanwhile, there were no significant changes in nuclear immunofluorescence of p53 protein (Figure 8; control (c1) and (c2) versus LPS (d1) and (d2)). 

**Figure 9:**
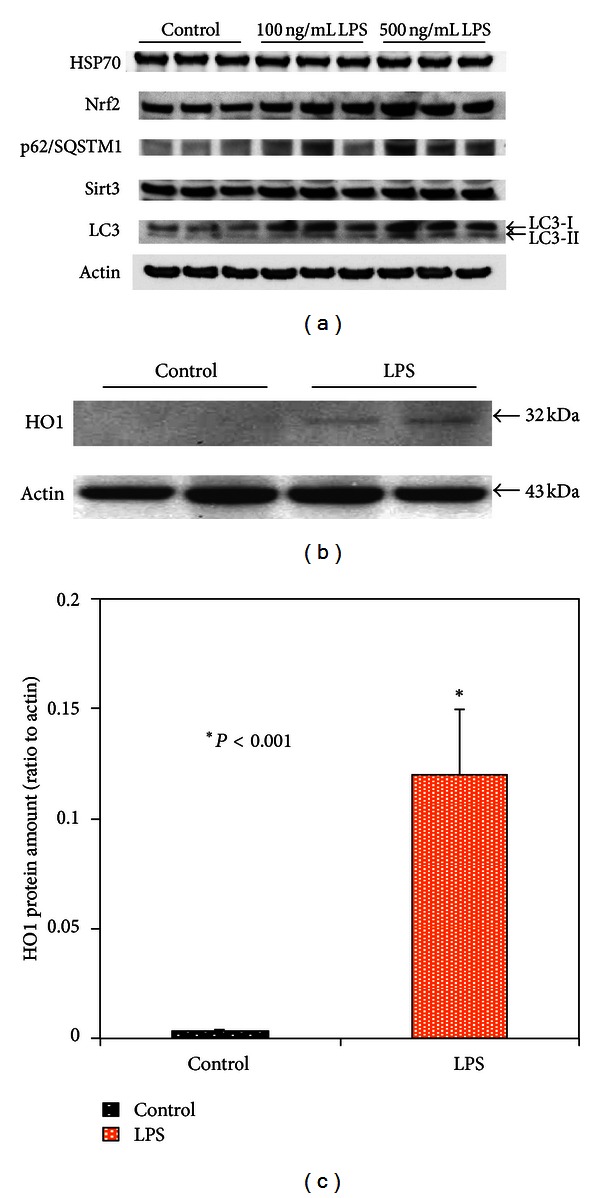
Western immunoblot analysis of redox-response and autophagy-mediated proteins in MSCs challenged with LPS. (a) Representative immunoblotting bands of HSP70, Nrf2, p62/SQSTM1, Sirt3, and LC3 proteins. The protein extracts were obtained from MSC cultures 24 h after challenge with LPS. ((b)-(c)) Representative immunoblotting bands of Hemeoxygenase 1 (HO1) protein (b) and respective densitometry histograms of HO1 bands (c) in MSCs stimulated with LPS. The presented bars indicate the relative density of HO1 protein (normalized to density of actin bands). The statistical significance was determined by Student's *t*-test (*n* = 3). Conditions: MSCs were incubated with 500 ng/mL LPS for 3 h. The cells were harvested at 24 h following challenge with LPS.

The above analyzed transcriptional factors, that is, Nrf2, NF*κ*B, and FoxO3a, are implicated in regulation of a variety of adaptogens, antioxidants, and mediators of autophagy and mitochondrial remodeling including HSP70, HO1, p62, Sirt3, and LC3 [23, 24, 33, 35, 42]. Moreover, a growing body of evidence suggests involvement of chaperone heat-shock proteins and adaptor proteins in autophagy events [42, 43]. The results of immunoblot analyses of these proteins in the LPS-treated MSCs are shown in Figure 9(a). LPS-induced expression of HSP70 and Sirt3 was insignificant (Figure 9(a)) and was apparently due to high levels of the constitutively present proteins. Meanwhile, we observed a substantial increase in HO1 protein (Figure 9(b)) that was in concord with the LPS-induced response of Nrf2 (Figures 7 and 9(a)), a transactivator of HO1 [35]. 

Remarkable responses occurred in the ubiquitin-associated target adaptor p62/SQSTM1 and LC3 type I and type II proteins (Figure 9(a)), which are mediators of macroautophagy (ATPhG) [11, 22, 37, 43]. A key step in the autophagosome biogenesis is the conversion of light-chain protein 3 type I (LC3-I, also known as ubiquitin-like protein, Atg8) to type II (LC3-II). The conversion occurs via the cleavage of the LC3-I carboxyl terminus by a redox-sensitive Atg4 cysteine protease. The subsequent binding of the modified LC3-I to phosphatidylethanolamine, that is, the process of lipidation of LC3-1, on the isolation membrane as it forms, is mediated by E-1- and E-2-like enzymes Atg7 and Atg3 [11, 22, 37]. Thus, conversion of LC3-I to LC3-II and formation of LC3-positive vesicles are considered to be a marker of activation of ATPhG [11, 22, 37]. As shown in Figure 9(a), a challenge of MSC with LPS resulted in increases in both LC3-I and LC3-II expression as determined by immunoblotting and indicated upregulation of the LC3-I to LC3-II transition. At this stage, our further investigation was focused on immunofluorescence confocal imaging and TEM analysis of ATPhG-mediated remodeling in the LPS-challenged cells.

In the second set of experiments, we analyzed autophagy/autolysosomal response and mitochondrial remodeling in MSCs subjected to LPS challenge. The ATPhG pathway is considered to be an evolutionarily developed prosurvival mechanism, which removes and processes damaged and misfolded proteins, and compromised organelles in response to redox stress [21, 25, 27, 37, 44]. Activation of ATPhG is associated with formation of autophagic/autolysosomal vacuoles in the cytoplasm which mediate proteolytic processes [11, 22, 25, 27].

**Figure 10:**
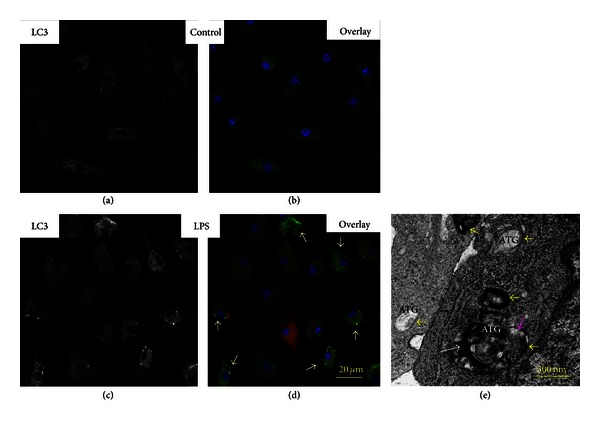
Assessment of autophagosome formation in MSCs challenged with LPS. ((a)–(d)) Confocal immunofluorescence imaging. (a) Green channel. LC3 projection in control MSCs. (b) Overlay of projections of LC3 (green channel), iNOS (red channel), and nuclei (blue channel) in control MSCs. (c) Green channel. LC3 projection in MSCs challenged with LPS. (d) Overlay of projections of LC3 (green channel), iNOS (red channel), and nuclei (blue channel) in MSCs challenged with LPS. Spatial localization of LC3 is indicated with white arrows. Conditions: MSCs were incubated with 500 ng/mL LPS for 3 h. The cells were analyzed 24 h after challenge with LPS. Counterstaining of nuclei was with Hoechst 33342 (blue channel). The confocal images were taken with pinhole setup to obtain 0.5 *μ*m Z-sections. (e) TEM image of MSCs challenged with LPS. Autophagosome (ATG) membranes are indicated with yellow arrows; fragments of mitochondrion in ATG are indicated with white arrow; fusion of lysosomes with ATG is indicated with pink arrow.

The images presented in Figure 10 indicate that upregulation of LC3-I/LC3-II proteins in the LPS-challenged cells was associated with massive formation of the LC3-positive vesicles featuring autophagosomes and autolysosomes.

**Figure 11:**
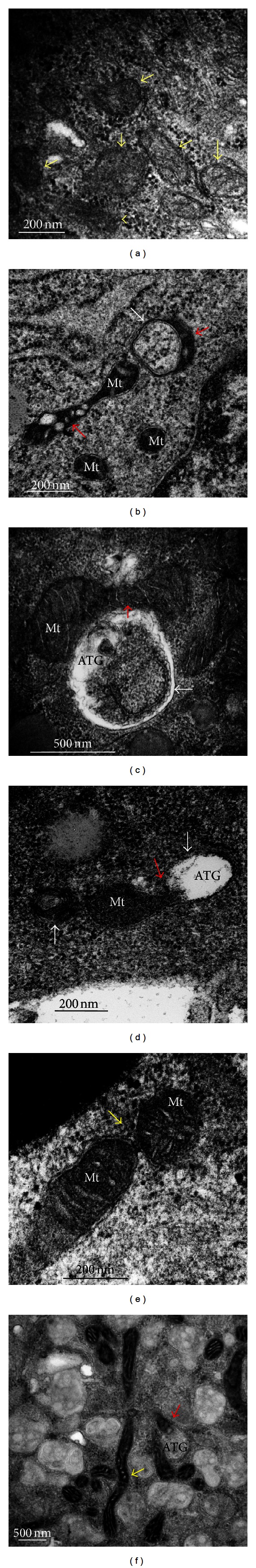
Transmission electron (TEM) analysis of mitochondrial remodeling inMSCs challenged with LPS. (a) Image of a control MSC. Mitochondria are indicated with yellow arrows. ((b)–(f)) Images of MSCs challenged with LPS. (b) Damaged mitochondria subjected to remodeling and mitophagy are shown with red arrows. Double-layer membrane of an autolysosome is indicated with white arrow. Mt, mitochondria. ((c)-(d)) Fusion of damaged mitochondria (Mt) with autophagosomes (ATG) is indicated with red arrows. ATG membranes are indicated with white arrows. (d) Formation of secretory autolysosomes containing multilamellar structures (indicated with red arrows) in an irradiated MSC. (e) Fusion of mitochondria (Mt) is indicated with yellow arrow. (f) Formation of elongated mitochondria is indicated with white arrow. Mitophagy is indicated with red arrow. Conditions: MSCs were incubated with 500 ng/mL LPS for 3 h. The cells were analyzed 24 h after challenge with LPS.

The further assessment of autophagy events with TEM revealed in the LPS-challenged cells the presence of characteristic multiple vacuoles, which were formed by double-layer membranes and sequestered constituents of different densities (Figure 10). Some of these vacuoles can be identified as secretory autolysosomes by the presence of multilamellar structures (most likely fibers of collagen) released extracellularly, while others contained fractured organelles including compromised mitochondria (Figure 11). 

Recent observations suggest that autophagosomes do not form randomly in the cytoplasm but rather sequester mitochondria selectively [45, 46]. Selection of compromised mitochondria for mitophagy requires activation of the PINK1/PARKIN pathway and implication of adaptor proteins, for example, p62/SQSTM1, and ubiquitin-like modifiers, which target mitochondria and ultimately mediate fusion of the processed mitochondria with autophagosome [45, 46]. 

The images presented in Figures 10(e) and 11(b)–11(d) indicate that mitochondria can be fused with autophagosomes in similar sizes while further degradation occurred in large-size autolysosomes.

**Figure 12:**
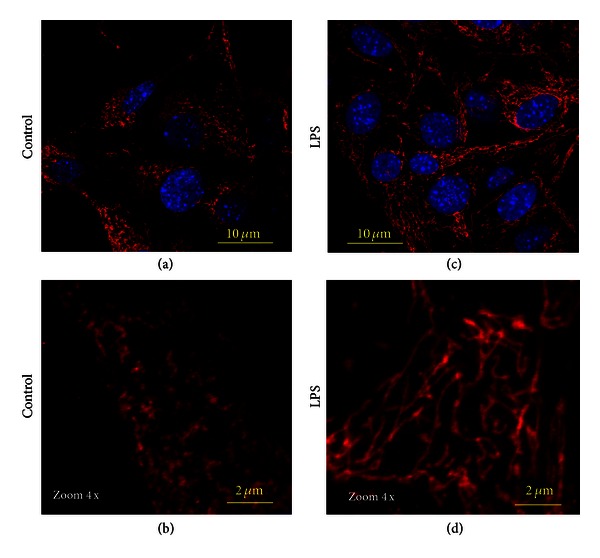
Confocal immunofluorescence imaging of mitochondrial remodeling in MSCs challenged with LPS. Mitochondrial networks were visualized using projections of TOM20 (red channel), a mitochondrial marker. ((a)-(b)) Control MSCs: mitochondrial network is presented by small-size dots. ((c)-(d)) MSCs challenged with LPS: formation of long-length mitochondrial network occurred due to mitochondrial fusion. Conditions: MSCs were incubated with 500 ng/mL LPS for 3 h. The cells were analyzed 24 h after challenge with LPS. Counterstaining of nuclei was with Hoechst 33342 (blue channel). The confocal images were taken with pinhole setup to obtain 0.5 *μ*m Z-sections.

The observed mitophagy was accompanied by extensive mitochondrial fusion resulting in the remodeling and expansion of the mitochondrial network (Figures 11 and 12). The events of mitochondrial fusion and formation of elongated mitochondria (over 10 *μ*m length) were captured with TEM and confocal immunofluorescence microscopy and are presented in Figures 11(e), 11(f), and 12(d). All the above data suggest that a short-term challenge with LPS triggered in MSCs a battery of complex adaptive responses leading to an increase in resistance to redox stress and damage to cellular constituents, and also to the remodeling of the entire mitochondrial network. 

## 4. Discussion

The redox stress occurs in the pathogeneses of a variety of injury types, including radiation combined injury and the associated sepsis, and there are multiple original papers and reviews that were addressed recently to this phenomenon [4, 14, 47–49]. The associated secondary oxidative injury to sensitive cellular constituents and ER stress can affect homeostasis of tissue barriers and thus exacerbate the process of healing. With this respect, development of new regiments for treatment of complicated injuries can be made more efficient with better understanding of the basic cellular mechanisms implicated in redox adaptive responses in tissue barriers. This particular area of the molecular redox pathophysiology is poorly developed despite the fact that the general concept of cellular stress responses is broadly discussed in the literature [35, 38, 50]. Our communication is the first report demonstrating a potential role of MSCs in sustaining redox resistance of barrier functions under septic conditions.

According to the current paradigm, general stress responses involve conserved signaling modules that, in turn, are interconnected to the cellular adaptive mechanisms [21, 33, 50]. It has been shown recently that bacterial infections trigger specific sensitive mechanisms mediating inflammation, redox stress, adaptation, and remodeling [2, 3, 17, 20–22]. Redox stress *per se* stimulates signaling cascades mediated by transcription factors and pathways that are believed to play a central role in cell survival. These include, but are not limited to, a battery of thiol-containing redox-response elements, redox-sensitive transcription factors such as nuclear factor-kappa B (NF*κ*B), Nrf2, FoxO3a, and stress-response adaptors such as the chaperone heat-shock protein 70 (HSP70) and NAD^+^-dependent deacetylase sirtuin-3 (Sirt3), and activators of the autolysosomal degradation and mitochondrial remodeling. Overall, these effector systems are crucial in maintaining homeostasis, which is altered due to oxidative damage to cell constituents [35–46]. It should be noted that, while the role of the redox-induced NF*κ*B and Nrf2 responses in cell survival is well documented, transcriptional factor FoxO3a, the autophagy/autolysosomal pathway, and mitochondrial remodeling are relatively newly determined players implicated into adaptive mechanisms [21, 30, 31, 36, 37, 42–46]. 

Recently, we demonstrated *in vitro* that MSCs could employ ATPhG in phagocytosis of *Escherichia coli* [11]. However, a mechanism which allowed the cells to avoid the adverse effects of the products of bacterial biodegradations, such as LPS, remained unclear. The data presented in this report indicate that *in vitro* challenge of MSCs with LPS inflammagen triggered a cascade of responses that we believe orchestrate adaptive remodeling of the cell and increase resistance to a “self-inflicted” LPS-induced oxidative stress. A pattern of these adaptive responses include induction of redox-response elements such as NF*κ*B, TRX1, Ref1, Nrf2, FoxO3a, and activation of ATPhG and mitochondrial remodeling. It should be noted that despite the presence of mitophagy and mitochondrial remodeling in the LPS-challenged MSCs, there were no significant alterations in levels of Sirt3 protein, which was a major player in mitochondrial response to the redox stress [35, 37, 46]. We assumed that it was likely due to a high constitutive expression of this protein in MSCs. Meanwhile, unlike Sirt3, there occurred a significant expression of HO1, an antioxidant protein, which utilized mitochondrial heme, which *per se* is a catalyst of the Fenton type of reactions as well as being essential for the *de novo* formation of active form of iNOS [35, 51]. Overall, our observations support a general concept of the presence of a network of a variety of signaling pathways that enable mediating cellular adaptation to the oxidative stress [33, 35, 37, 40, 43, 50].

## Figures and Tables

**Table 1:** qRT-PCR assessment of gene transcription in MSCs challenged with LPS.

LPS	IL1A	IL1*β*	IL6	IL8	iNOS
ng/mL	Expression, a.u.	Expression, a.u.	Expression, a.u.	Expression, a.u.	Expression, a.u.
0	1 ± 3.25	1 ± 1.82	1 ± 1.54	1 ± 1.41	1 ± 3.14
50	194 ± 2.77*	317 ± 1.63*	99 ± 1.48*	26 ± 1.8*	882 ± 2.46*
100	265 ± 2.33*	465 ± 1.78*	155 ± 1.4*	46 ± 1.36*	1128 ± 2.3*
500	315 ± 2.35*	755 ± 1.68*	47 ± 2.67*	18 ± 1.98*	1596 ± 2.46*
1000	247 ± 2.38*	498 ± 1.78*	98 ± 1.6*	11 ± 1.61*	1489 ± 2.37*
2500	338 ± 2.35*	729 ± 1.57*	224 ± 2.32*	20 ± 3.33*	1438 ± 2.25*

Conditions: MSCs were incubated with 50 ng/mL–2500 ng/mL LPS for 3 h and then were lysed for mRNA extraction and qRT-PCR analysis. The presented data are statistically significant at the confidence level *P < 0.005, (*n* = 3).
